# Home Literacy Environment and Children’s English Language and Literacy Skills in Hong Kong

**DOI:** 10.3389/fpsyg.2020.569581

**Published:** 2021-01-29

**Authors:** Carrie Lau, Ben Richards

**Affiliations:** Faculty of Education, The University of Hong Kong, Pokfulam, Hong Kong

**Keywords:** home literacy environment, English as a second language, language and literacy, early years, parent-child engagement

## Abstract

Emerging evidence has shown a positive association between the home literacy environment (HLE) and monolingual children’s language and literacy development. Yet, far fewer studies have examined the impact of the HLE on second language development. This study examined relations between the HLE and children’s development of English as a second language in Hong Kong. Participants were 149 ethnic Chinese children (80 girls; *M*_age_ = 59 months, *SD*_age_ = 10 months) and one of their caregivers. Caregivers completed questionnaires about their family backgrounds and HLE and children were assessed on their English language and literacy skills. Findings revealed considerable variability in the types of literacy activities that caregivers were engaged in at home with their children. A series of multilevel regressions demonstrated that the HLE was differentially associated with English vocabulary, letter knowledge, phonological awareness, and word reading skills after controlling for child and family characteristics. Results highlight the importance of a literacy-rich home environment for children’s development of English as a second language and the need to support caregivers in providing a range of home literacy activities to facilitate different language and literacy skills.

## Introduction

It has been well documented that differences in language and literacy skills emerge early in life (Hart and Risley, 1995; Fernald et al., 2013). Extant research has explored individual and environmental factors that underlie variability in language growth and development (Dickinson and Tabors, 2001). Mounting evidence suggests that the home literacy environment (HLE) is one of the most significant predictors of early language and literacy development (Frijters et al., 2000; Niklas et al., 2015). As gaps in language, literacy and achievement persist over time and can have long-lasting impact on children (Stanovich, 1986), it is critical to understand the characteristics and role of the HLE starting from the early years in order to disentangle the factors and processes associated with language and literacy outcomes and to identify the kinds of support needed for children and families.

With the rise of English as a global language (Crystal, 2012), a growing number of studies has investigated the association between the HLE and children’s development of English as a second language. However, to date, most studies that examined the influence of the HLE on children’s proficiency in English as a second language have primarily been conducted in predominantly English-speaking contexts (e.g., Duursma et al., 2007; Farver et al., 2013). Far less research has focused on HLE and second language learners of English in multilingual contexts (e.g., Kalia and Reese, 2009; Dixon, 2011). Against this background, the present study examined whether and how HLE is associated with the development of English as a second language in a sample of ethnic Chinese children from Hong Kong.

## Conceptualizing the Home Literacy Environment

Much of the early research on the HLE primarily focused on differences in HLE by family socioeconomic background (e.g., income and parental education) or on a single literacy activity, most notably parent-child reading as a defining feature of the HLE (Payne et al., 1994; Scarborough and Dobrich, 1994). Later work conceptualized the HLE as a multidimensional construct that encompassed a variety of literacy-related interactions, resources and attitudes, consisting of parent-child joint activities, such as shared reading, parental teaching of print-related skills, singing songs and rhymes, storytelling and watching educational television programs (Frijters et al., 2000; Wood, 2002); availability of learning materials, such as the number of books at home (Sénéchal et al., 1998); and parental beliefs and attitudes toward literacy (Debaryshe, 1995; Weigel et al., 2006). Based on the Home Literacy Model (Sénéchal and LeFevre, 2002; Sénéchal, 2006), home literacy experiences can be categorized into formal and informal interactions. Formal literacy interactions refer to activities in which the focus is on the features of print (e.g., adults directly teaching children print-related skills, such as letter names and sounds; adults pointing to letters in the text), whereas informal literacy interactions refer to opportunities that are centered on the meaning attached to print (e.g., often manifested by shared reading; adults focusing on meaning carried by the text during shared reading). The HLE can be further differentiated into active components, which emphasize parent-child engagement in literacy activities and passive components, which refer to children’s observations of parents modeling literacy behaviors (e.g., parents’ engagement in reading) (Burgess et al., 2002).

## Home Literacy Environment and Children’s Language and Literacy Outcomes

An extensive body of research has shown concurrent and longitudinal links between the HLE and children’s early language and literacy development (Burgess et al., 2002; Manolitsis et al., 2011; Rodriguez and Tamis-Lemonda, 2011; Sénéchal and LeFevre, 2014; Tamis-Lemonda et al., 2019). Shared reading –the most studied aspect of the HLE—has been found to contribute significantly to the development of receptive and expressive vocabulary (Sénéchal et al., 1998; Evans and Shaw, 2008; Farrant and Zubrick, 2012), letter name and letter sound knowledge (Frijters et al., 2000), and as well as listening comprehension (Sénéchal and LeFevre, 2002). In several meta-analytic reviews (e.g., Scarborough and Dobrich, 1994; Bus et al., 1995; Mol et al., 2008, 2009), the frequency of exposure to parent-child reading accounted for unique variance in children’s language and literacy skills, and later reading achievement. Other indices of shared reading, such as the number of books in the home, visits to the library, children’s requests to be read to and the age at which children were first read to by their parents contributed substantial variance to language growth (Debaryshe, 1993; Payne et al., 1994; Raikes et al., 2006). The quality of book reading, including the reading behaviors of parents and interactions during shared reading was also found to be significant correlates of children’s language and literacy outcomes (van Kleeck et al., 1997; Deckner et al., 2006). Correlational (e.g., Haden et al., 1996) and intervention studies (e.g., Whitehurst et al., 1988; Reese and Cox, 1999; Justice and Ezell, 2000) revealed that reading behaviors, such as asking questions, labeling and describing objects, and providing feedback and focusing on print yielded significant positive effects on vocabulary and print knowledge.

Another aspect of the HLE, direct teaching of print-related skills (e.g., letter recognition and letter sounds) has been found to predict children’s alphabet knowledge (Evans et al., 2000; Hood et al., 2008; Hindman and Morrison, 2012; Martini and Sénéchal, 2012), phonological awareness (Foy and Mann, 2003; Johnson et al., 2008; Niklas and Schneider, 2013), word reading (Puglisi et al., 2017), and writing (Puranik et al., 2018). Existing studies have combined a range of parent-child joint activities as a measure of the HLE in predicting early language and literacy outcomes (e.g., Griffin and Morrison, 1997; Leseman and de Jong, 1998; Wood, 2002; Van Steensel, 2006). For instance, Van Steensel (2006) found that children who participated in a variety of joint literacy activities, such as shared reading, library visits and singing nursery songs, as well as observed parents and/or siblings engaging in literacy activities themselves exhibited gains in vocabulary and general reading comprehension. Wood (2002)’s study demonstrated that children who were exposed to four types of parent-child literacy activities (i.e., storybook reading, letter-based activities, singing, and playing language games) had significantly higher vocabulary and reading ability scores as compared to their counterparts who were engaged in singing only or to those that did not participate in almost any of the literacy activities. Weigel et al. (2006)’s findings revealed that children’s engagement in a variety of parent-child joint activities, such as shared reading, storytelling, singing rhymes, drawing pictures, playing games and television viewing was associated with enhanced print knowledge. Indeed, several large-scale longitudinal studies have adopted a multidimensional approach in examining the HLE that captures variations in the type of literacy activities that children are exposed to at home. For instance, the Index of Early Home Literacy Activities of the Progress in International Reading Literacy Study (Mullis et al., 2007) examines early literacy experiences through six activities, namely reading books, telling stories, singing songs, playing with alphabet toys, playing word games and reading aloud signs and labels.

Cumulative research has demonstrated the associations between aspects of the HLE and children’s early language and literacy skills in their native language (Sénéchal et al., 1998; Hindman and Morrison, 2012). Studies with children from different ethnic backgrounds and/or contexts who are learning English as a second or foreign language have found similar results (e.g., Hammer et al., 2003; Duursma et al., 2007; Kalia and Reese, 2009). Farver et al. (2013)’s study with Latino immigrant children in the United States found that parents’ engagement in activities was positively associated with children’s oral language skills in both English and Spanish. Further, home literacy resources in English and parents’ literacy behaviors in Spanish were associated with children’s print knowledge in both English and Spanish. In another study with Indian children learning English, it was found that book reading practices and parental teaching predicted children’s print skills in English and that book reading practices moderated the relationship between the degree of English spoken at home and children’s English receptive vocabulary skills (Kalia and Reese, 2009). Indeed, as there is greater complexity in the HLE of children and families that navigate multiple languages in their homes and community contexts, it is worthwhile to identify specific pathways through which the HLE may impact children’s language and literacy development. Oral language and early literacy skills are interrelated components that provide a crucial basis for children’s academic success and subsequent educational attainment in school. In the development of English, vocabulary, phonological awareness, and letter knowledge have been found to predict word reading abilities among first and second language learners (Whitehurst and Lonigan, 1998; Muter et al., 2004). This study therefore, explores the relationship between a combination of home literacy activities and the development of English as a second language among ethnic Chinese children in Hong Kong and focuses specifically on English vocabulary, phonological awareness, letter knowledge, and word reading skills.

## The Hong Kong Context

Hong Kong was a British colony from 1841 to 1997. During most of the colonial period, English was the sole official language. In 1974, Chinese became a co-official language alongside with English. Since 1997, the Hong Kong government has adopted the “biliterate and trilingual” language policy to enable its citizens to become proficient in written English and Chinese and in spoken English, Cantonese, and Putonghua. Cantonese is the mother tongue of the majority of the local population and is used most often in workplace and non-workplace settings, such as in the communication with spouses, children, parents, friends, colleagues, and clients (Bacon-Shone et al., 2015; Census and Statistics Department HKSAR, 2019). Over time, the proportion of the population using English as the usual language (i.e., in daily communication) increased from 2.2% in 2001 to 4.3% in 2016 (Census and Statistics Department HKSAR, 2017). Among individuals with children aged six and below and whose mother tongue is not English, around 13.7% must or often use English to communicate with their children. The most cited reasons for parents to use English were to offer children the opportunities to be exposed to English and the belief that it is better for their children to learn English earlier (Census and Statistics Department HKSAR, 2019).

Despite the predominant use of Cantonese among the general population in Hong Kong, proficiency in English is highly prized and is viewed as a vehicle for upward social mobility (Evans, 2000). English is one of the languages used in the Government and in legal, professional and business sectors. In education, English is the medium of instruction in private universities and in six out of eight government-funded universities (Kirkpatrick and Liddicoat, 2017). The outpouring of criticism of the compulsory Chinese medium instruction policy for secondary schools in 1998 eventually led to the fine-tuning of the medium of instruction policy in 2010 (Tollefson and Tsui, 2018), reflecting the priorities placed on English language education by stakeholders such as parents and the business community. Owing to the market-driven nature of the kindergarten sector in Hong Kong, parental preference for English further contributed to the push toward the early provision of English language teaching in schools amidst the implementation of the ‘biliterate and trilingual’ language policy (Leung et al., 2013).

English is promoted as a second language in the local school curriculum starting from the early years. As recommended in government curriculum and policy documents, English is introduced in kindergartens on condition that teachers possess appropriate levels of language proficiency and adopt an informal teaching approach, such as through the use of songs, storytelling, and language activities (Education Department, 1994, 1999; SCOLAR, 2003). The objectives of English language teaching in the early years are to nurture children’s interest, attitude and confidence toward English and to develop basic skills, such as understanding simple conversations and words (Curriculum Development Council, 2006, 2017). The frequency and structure of English language teaching, however, vary considerably across kindergartens such that English is: (i) taught as a subject by local and/or native English-speaking language teachers (i.e., children are exposed to the language for only a certain amount of time per week); (ii) introduced within a bilingual/trilingual program (i.e., children are simultaneously exposed to multiple languages during the school period and an English class teacher may be present alongside a Cantonese and/or Putonghua class teacher in the classroom); or (iii) used as the main medium of instruction (Lau and Rao, 2013; Ng and Rao, 2013). In Hong Kong, kindergartens are categorized as either private-independent or non-profit making. The latter makes up 80% of all kindergartens in Hong Kong (Education Bureau, 2019) and are eligible to apply for the Kindergarten Education Scheme (in which kindergartens are funded by the government to provide free half-day services for children) (Education Bureau, 2016). In most non-profit making kindergartens, Chinese (Cantonese) is the medium of instruction and English is taught as a subject (Lau, 2020). The variation in exposure to English, coupled with differences between the first and second language (Chinese as a morphosyllabic language versus English orthography), poses some unique challenges for children in learning English in Hong Kong.

To date, only limited empirical studies have examined factors and contexts that underlie English language development among young children in Hong Kong. A small number of studies have specifically explored the HLE and children’s English language and literacy development (e.g., Yeung and King, 2016; Tse et al., 2017). In Yeung and King’s (2016)’s study of the HLE among children learning English as a second language, it was found that there were variations in home support and parents were engaged in home teaching (e.g., homework instruction) more frequently than in shared reading and in the provision of literacy materials. Findings revealed differential impacts of the HLE on children’s English language and literacy outcomes. Shared reading predicted children’s receptive and expressive vocabulary, syllable awareness and word reading skills while home teaching predicted letter knowledge and the provision of literacy materials predicted expressive vocabulary only. Tse et al. (2017) further demonstrated the long-term impact of early home reading activities (i.e., prior to entry into primary school) on the Chinese and English reading attainment of 1376 Grade 4 students. Specifically, a combination of activities including storybook reading, storytelling, singing songs, playing word games, writing letters and reading aloud signs contributed to children’s reading performance in English. However, it was noted that a sizeable number of parents never or almost never engaged in home reading activities in English prior to or during their children’s primary schooling. Related studies point to the role of family processes in children’s school readiness in Hong Kong. Lau et al. (2011) found that parents were engaged more in home-based involvement than in school-based involvement. Home-based involvement, including the provision of language and cognitive activities had the strongest predictive relationship to children’s school readiness. In another study, Ip et al. (2016) demonstrated that reading (e.g., storybook reading and storytelling) and recreational activities (e.g., listening to music and playing together) in the home learning environment significantly mediated socioeconomic gradients in children’s school readiness. Intervention studies on parent-child reading also revealed positive effects on children’s language and literacy development. Chow et al. (2010)’s study demonstrated the effectiveness of a 12-week parent-child reading intervention (dialogic reading vs. typical reading vs. control) on children’s development of English as a second language. More specifically, both typical reading and dialogic reading yielded significant intra-group gains in word reading skills. Further, children in the dialogic reading condition had gains in phonological awareness. Together, these studies suggest the importance of parental engagement at home and the provision of a literacy-rich environment to support children’s development.

However, much remains unknown about the types of home literacy activities that caregivers are engaged in to support children’s English language learning, as well as the potential role of related factors in children’s development of English as a second language in Hong Kong. The current study extends previous research by examining the HLE more extensively (e.g., including reading behaviors and media-based activities) in relation to a range of early English language and literacy skills. Further, this study considers a host of factors that underlie children’s exposure to English (e.g., enrolment in extracurricular English lessons, amount of English exposure at school) in the analysis of the predictive role of the HLE on early English language and literacy outcomes. The present work is situated within theoretical frameworks that highlight the interactions and interrelationships among individual and environmental factors and is underpinned by: (i) the bioecological theory that views home experiences as proximal processes that serve as primary engines in predicting child development (Bronfenbrenner and Morris, 2006); (ii) the social learning theory, which stresses the role of interactions with more experienced others, such as parents in optimizing development and learning (Vygotsky, 1978); and (iii) the attachment theory, which highlights the significance of responsive, stimulating and supportive caregiving in child development (Bretherton, 1985). Arising from the aforementioned theories is also the notion that culture plays an integral part of proximal processes that shape children’s development, including language and thought. Hong Kong is uniquely positioned for the study of the HLE amidst culturally specific parenting values and practices among Chinese parents (e.g., priorities on academic preparedness) (Luo et al., 2013), the implementation of the “biliterate and trilingual” language education policy (Wang and Kirkpatrick, 2019) and the status of English in a post-colonial society (Bolton, 2012). Findings from this study will provide important insights into the nuances and complexities of the contextual support for English language learning in a multilingual context and enable the identification of specific dimensions of the HLE that effectively facilitate the development of English as a second language among young learners. The research questions for this study were as follows: (a) What kinds of home literacy practices are caregivers engaged in to support children’s English language and literacy development?; (b) What is the relationship between the HLE and children’s English language and literacy skills?; (c) To what extent does the HLE predict children’s English language and literacy skills? Based on the review of learning-related practices of Chinese parents in Chinese contexts (Ng and Wei, 2020), it is hypothesized that caregivers will engage more in direct teaching of print-related skills than in other home literacy activities, such as shared reading. The HLE, as measured by caregivers’ reports of their engagement in literacy activities with children, will be positively associated with early English language and literacy outcomes even when controlling for child and family characteristics. It is expected that different aspects of the HLE will be differentially related to children’s English language and literacy skills. Specifically, based on Chow et al.’s (2010) findings, it is expected that shared reading will be associated with a range of English language and literacy skills.

## Materials and Methods

### Participants

A total of 149 children (69 boys and 80 girls) between the ages of 39 and 81 months (*M*_age_ = 59 months, *SD*_age_ = 10 months) and one of their caregivers were recruited from one K1 (for 3- to 4- year olds), K2 (for 4- to 5- year olds) or K3 (for 5- to 6- year olds) class from 10 non-profit making kindergartens in Hong Kong. The number of children recruited from each kindergarten ranged from 8 to 26. Information on the frequency and content of English language teaching was collected through an interview with the English teacher in the participating kindergartens. English language teaching ranged from 20 to 40 min per session and from 1 to 5 days per week. The curricula and teaching contents in English language teaching were comparable across kindergartens and emphases were placed on the development of letter knowledge and sounds, vocabulary and sentence structures through a variety of activities, such as storybook reading, singing songs, playing word games, and pre-reading and pre-writing opportunities.

Table 1 shows descriptive statistics of children and their families. Participating caregivers were mostly the child’s mother (81%) or father (17%), whilst 2% were other caregivers. Caregivers provided demographic and socioeconomic information about both parents using a questionnaire. All children in the sample were exposed to English lessons at school, ranging from 0.7 h per week to 2 h per week. Parents’ highest educational qualification was measured over 7 levels from primary education to doctoral degree. The mean highest qualification for both mothers and fathers was close to level 3 (upper secondary), with a mean of 3.1 for mothers and 3.2 for fathers. Household income was measured across 10 bands, from less than $4,000 HKD per month to greater than or equal to $100,000 HKD per month. The mean (4.8) was close to band 5 ($30,000 to $39,999 HKD per month). Respondents reported the primary language(s) used at home, with 95% of respondents using Cantonese, 20% using Mandarin, 15% using English, and 2% using another language (respondents could select all options that applied). Twenty-three percent of children had extracurricular English lessons.

**TABLE 1:** Descriptive statistics of key variables for children in the sample.

	***n***	**Mean (%)**	**SD**	**Minimum**	**Maximum**
**Background variables**					
Age (months)	149	59.29	9.69	39.80	80.63
Non-verbal IQ	149	6.89	2.03	2.00	11.00
Gender (% girls)	149	54			
English primary language at home (%)	148	15			
Extracurricular English lessons (%)	149	23			
Hours of English at school per week	149	1.13	0.45	0.67	2.00
Mother’s highest qualification (7 levels)	148	3.15	1.21	1.00	6.00
Father’s highest qualification (7 levels)	146	3.23	1.30	1.00	6.00
Household income (10 income bands)	148	4.81	1.98	2.00	10.00
**Language and literacy measures**					
Phonological awareness	149	1.63	2.22	0.00	10.00
Receptive vocabulary	149	16.97	4.61	7.00	24.00
Letter knowledge	149	18.06	7.60	1.00	26.00
Word reading	149	4.25	8.03	0.00	30.00

*Descriptive statistics are shown before imputation of missing values and before variable standardization. There were 7 levels of qualifications: Primary; Lower Secondary (Grade 7–9); Upper Secondary (Grade 10–12); Higher Certificate, Diploma, or Associate Degree; Bachelor’s degree; Master’s degree; and Doctoral Degree. Income was measured as household monthly income in HKD, across 10 bands: under $4,000; $4,000– $9,999; $10,000 – $19,999; $20,000 – $29,999; $30,000– $39,999; $40,000– $49,999; $50,000– $59,999; $60,000– $79,999; $80,000– $99,999; and ≥ $100,000.*

### Measures

Children were directly assessed using one measure of non-verbal intelligence, and four measures of English language and literacy: receptive vocabulary, phonological awareness, letter knowledge, and word reading. The HLE was measured based on responses to the caregiver questionnaire. Socio-demographic variables were also created based on responses to the caregiver questionnaire. School information on English language teaching was obtained through the teacher interview.

#### Non-verbal Intelligence

Sets A and B (24 items) of the Raven’s Colored Progressive Matrices (Raven et al., 1995) were administered to assess children’s non-verbal intelligence. Children were asked to select one missing piece from six available options to complete a matrix-like pattern with a missing section. One point was awarded for every correct answer. Cronbach’s alpha was 0.58.

#### Receptive Vocabulary

Two item sets (24 items) of the Peabody Picture Vocabulary Test- IV (PPVT-4; Dunn and Dunn, 2007) were used to measure children’s receptive vocabulary. Children were presented with four pictures and asked to point to the illustration that corresponded to the word that was orally presented by the assessor. One point was awarded for every correct answer. As the PPVT-4 was not normed within the local Hong Kong population, raw scores were used in the analysis. Cronbach’s alpha was 0.84.

#### Phonological Awareness

The elision sub-test (20 items) of the Comprehensive Test of Phonological Processing – Second Edition (CTOPP-2) (Wagner et al., 2013) was used to measure children’s phonological awareness. The assessor read aloud a two-syllable word and children were asked to delete a target syllable (e.g., say airplane without saying plane) or to delete phonemes from each word that was presented orally by the assessor (e.g., say cup without saying/k/). One point was awarded for every correct answer. As the CTOPP-2 was not normed within the local Hong Kong population, raw scores were used in the analysis. Cronbach’s alpha was 0.76.

#### Letter Knowledge

Children were asked to name the lowercase letters of all 26 letters of the alphabet that were presented in random order. One point was awarded for every correct answer. Cronbach’s alpha was 0.95.

#### Word Reading

Children’s word reading skills were assessed using a locally developed test by McBride-Chang and Kail (2002). This test consisted of 30 English words that were constructed from textbooks used in kindergartens in Hong Kong. Children were presented with the English words and asked to read each word aloud. One point was awarded for every correct answer. Cronbach’s alpha was 0.98.

#### Home Literacy Environment

The caregiver questionnaire consisted of items that tapped into the frequency of caregivers’ engagement in English literacy activities with children, such as shared reading (e.g., number of children’s books, age at which the child was first read to, frequency of shared reading and parents’ reading behavior during shared reading), storytelling, direct teaching of print-related skills (e.g., letter sounds and alphabets), visiting the library, singing rhymes/songs, using apps or digital media, watching television programs and helping with schoolwork. The frequency of engagement was assessed on a 7-point likert scale ranging from 0 (never) to 7 (daily). Caregivers were also asked to indicate the extent to which they agreed with statements about their behaviors if and when they read to children (e.g., I emphasize printed words while reading) on a continuum from strongly disagree to strongly agree. Response choices for the number of children’s books in English were coded on a 7-point scale ranging from 0 (none) to 7 (more than 100). An overall composite variable representing the HLE was created by standardizing each item, taking the mean of all items, and standardizing the composite HLE variable.

#### Socio-Demographic Variables

The caregiver questionnaire also included items on child characteristics, as well as family demographic and socioeconomic information, such as household monthly income (10 levels), mother’s and father’s education level (highest educational qualification over 7 levels), the primary language(s) spoken at home, and whether or not children participated in extracurricular English lessons (see Table 1).

#### School-Level Data on English Language Teaching

Information about the structure and arrangement of English language teaching in each of the participating kindergartens was obtained through an interview with the English teacher in the participating child’s class. The interviewed teacher was asked about the duration and frequency of English language teaching per week, as well as the teaching content of the English curriculum at the school.

### Procedure

Written informed consent was obtained from kindergarten principals, teachers and parents. Caregivers completed a questionnaire to provide socio-demographic information about children and both parents. The questionnaires were distributed to caregivers and returned in sealed envelopes via children’s class teachers at the school. Children were individually assessed on their non-verbal intelligence and English language and literacy skills by trained assessors, who were undergraduates and graduates majoring in early childhood education. The assessments took place in a quiet area at the school and lasted around 20 to 30 min for each child. The English teacher in each of the participating class was interviewed about the structure and arrangement of English language teaching in the school. The interview lasted for about 5 min.

### Analytic Plan

All analyses were conducted using Stata 15.1. Descriptive statistics were calculated for the raw scores of each of the key variables. Composite variables representing receptive vocabulary, phonological awareness, letter knowledge, and word reading were calculated by summing the relevant items and standardizing the total.

Exploratory factor analysis (principal component factors) was conducted on all HLE items to explore the factor structure of the HLE measure. Loadings for each item were examined after orthogonal varimax rotation with the objective of attaining an optimal simple structure (Yong and Pearce, 2013). Variables were excluded if they had a high proportion of uniqueness or did not load onto a common factor, and the factor analysis was repeated. The result was the exclusion of three variables, and a final 3 factor solution explaining 75% of variance with 3 factors having eigenvalues greater than 1 (Table 2). A composite variable was created to represent each factor, based on the items that had high loadings (0.6 or above) on that factor. Each composite factor variable was calculated using the standardized mean of the items with high loadings and was then also standardized.

**TABLE 2:** Rotated 3 factor solution of Home Literacy Environment variables.

**Variable**	**Factor 1: Shared reading and storytelling**	**Factor 2: Teaching of print-related skills**	**Factor 3: Play and media-based activities**	**Uniqueness**
Age of child when first read to	**0.86**	0.11	0.19	0.22
Ask questions	**0.92**	0.12	0.15	0.12
Highlight or explain vocabularies	**0.93**	0.13	0.14	0.10
Emphasize printed words	**0.91**	0.09	0.09	0.15
Discuss sounds of words	**0.93**	0.17	0.10	0.09
Read English books	**0.81**	0.18	0.26	0.24
Tell stories in English	**0.70**	0.06	0.31	0.41
Sing English nursery rhymes/songs	0.29	0.16	**0.66**	0.46
Play with English digital media	0.27	0.14	**0.82**	0.24
Watch English TV programmes	0.20	0.21	**0.72**	0.40
Teach child English alphabet letters	0.19	**0.71**	0.46	0.25
Teach child letters sounds	0.18	**0.78**	0.22	0.32
Teach child to read English words	0.10	**0.83**	0.23	0.25
Teach child to write English words	0.19	**0.79**	−0.19	0.30

*The table shows the 3-factor solution (principal component factors) after orthogonal varimax rotation, explaining 75% of variance. Three factors had eigenvalues of greater than 1. Loadings of greater than 0.6 are in bold for ease of interpretation.*

A variable representing composite parental socioeconomic status (SES) was created using a latent factor measurement model using maximum likelihood estimation and allowing for missing values, based on the mother’s highest level of education, father’s highest level of education, and monthly household income. Correlations between all key variables were calculated (Table 3). Four OLS regressions were run, with each of receptive vocabulary, phonological awareness, letter knowledge, and word reading being the dependent variable in one of the four models, and age, non-verbal IQ, gender, whether English was the primary language at home, whether the child had experienced extracurricular English lessons, the composite SES variable, and the amount of English exposure at school included as control variables. Models were run twice, first without including the mean overall HLE independent variable, and then again whilst including the HLE independent variable. *R*^2^ values were noted in each case to examine model fit with and without the inclusion of the independent variable.

**TABLE 3:** Correlations between key variables.

	**PA**	**RV**	**LK**	**WR**	**Age**	**IQ**	**Gender**	**Eng. home**	**Extracur Eng.**	**Eng. school**	**SES**	**Overall HLE**	**HLE fac1**	**HLE fac2**	**HLE fac3**
Phonological awareness	1.00														
Receptive vocabulary	**0.40**	1.00													
Letter knowledge	**0.41**	**0.32**	1.00												
Word reading	**0.42**	**0.42**	**0.46**	1.00											
Age (months)	**0.41**	**0.21**	**0.39**	**0.36**	1.00										
Non-verbal IQ	**0.29**	**0.17**	**0.36**	**0.22**	**0.50**	1.00									
Gender (girl)	−0.06	0.09	−0.05	−0.09	0.03	−0.01	1.00								
English primary language at home	**0.19**	**0.34**	0.08	**0.18**	0.14	0.01	0.01	1.00							
Extracurricular English	0.13	**0.16**	0.15	0.09	**0.19**	**0.17**	−0.10	0.00	1.00						
Hours of English at school	0.06	**0.31**	0.06	**0.34**	**0.22**	0.05	0.08	**0.27**	0.07	1.00					
SES composite score	0.16	**0.26**	0.01	0.14	−0.04	−0.11	0.00	0.09	0.12	0.13	1.00				
Mean overall HLE	0.10	**0.40**	**0.19**	**0.21**	−0.07	−0.03	0.04	**0.26**	0.05	0.12	**0.19**	1.00			
Factor 1: Shared reading and storytelling	0.05	**0.35**	0.13	**0.20**	−0.10	0.00	0.08	**0.23**	0.03	0.11	**0.24**	**0.90**	1.00		
Factor 2: Teaching of print-related skills	0.09	**0.18**	**0.26**	0.11	0.11	0.00	0.10	0.14	0.06	0.06	−0.06	**0.63**	**0.29**	1.00	
Factor 3: Play and media-based activities	0.05	**0.31**	0.03	0.10	−**0.22**	−**0.17**	−0.09	0.15	0.01	−0.01	0.14	**0.70**	**0.46**	**0.46**	1.00

*Significant correlations (*p*s < 0.05) are shown in bold. Full variable names are shown in the left-hand row, and abbreviations of variable names are shown in the top column.*

Next, four separate random slope multilevel regressions were run, with each of receptive vocabulary, phonological awareness, letter knowledge, and word reading being the dependent variable in one of the four models, and the mean overall HLE variable being the independent variable in all four models. This procedure was then repeated three times, by replacing the overall HLE variable with the HLE variable representing factor 1, then factor 2, and then factor 3. The procedure was repeated once more, but this time with all three HLE factor variables included at the same time in each of the regression models. This process resulted in a total of 20 multilevel regressions. All models controlled for age, non-verbal IQ, gender, whether English was the primary language at home, whether the child had experienced extracurricular English lessons, the composite SES variable, and the amount of English exposure at school, and used kindergarten as the level 2 variable. Independent and dependent variables were standardized in all models. Non-verbal IQ, composite SES, and English exposure at school were also standardized, and age was recentered at its grand mean.

To check for the possibility that floor or ceiling effects might be biasing the results, sensitivity analysis was conducted using a Tobit regression model, which is capable of correct inference in cases where there are floor or ceiling effects (McBee, 2010). All regressions were run once more, this time using a mixed-effects Tobit model. Coefficient magnitudes between models were not directly comparable because it was necessary to use raw rather than standardized versions of each dependent variable. However, this procedure made it possible to check whether the direction (positive or negative) of any association, and the presence or absence of statistical significance, were consistent between the random slope multilevel regressions and the regressions using the Tobit model.

Missing values were found for maternal education (*n* = 1), paternal education (*n* = 3), and household income (*n* = 1), which were estimated as part of the calculation of the composite SES variable as described above. Missing values were also found for English as a primary language at home (*n* = 1), and this was imputed using multiple imputation. The mixed-effects Tobit regression function in Stata 15.1 does not support multiple imputation so this one case was dropped listwise for the Tobit models only. No other values were missing.

## Results

Descriptive statistics for the measures of children’s non-verbal intelligence and language and literacy skills (before standardization) are presented in Table 1. The HLE was measured based on questions from the caregiver questionnaire. Caregivers reported the number of English children’s books in the household. Six percent of caregivers reported having no books, 67% reported having between 1 and 20 books, and 27% reported having more than 20 books. Caregivers also reported the age at which their child was when they first started to have English read to them. Forty-one percent of caregivers stated that they did not read English to their child, 15% reported reading within the first 12 months, 15% reported reading within 13 and 23 months, and 29% reported starting reading English when their child was more than 2 years old. Of those that reported reading English to their child (*n* = 88), a majority of parents agreed that they asked questions (69%), highlighted or explained key vocabularies from the text (68%), emphasized printed words (68%), and discussed sounds of the words (66%) while reading.

**FIGURE 1:**
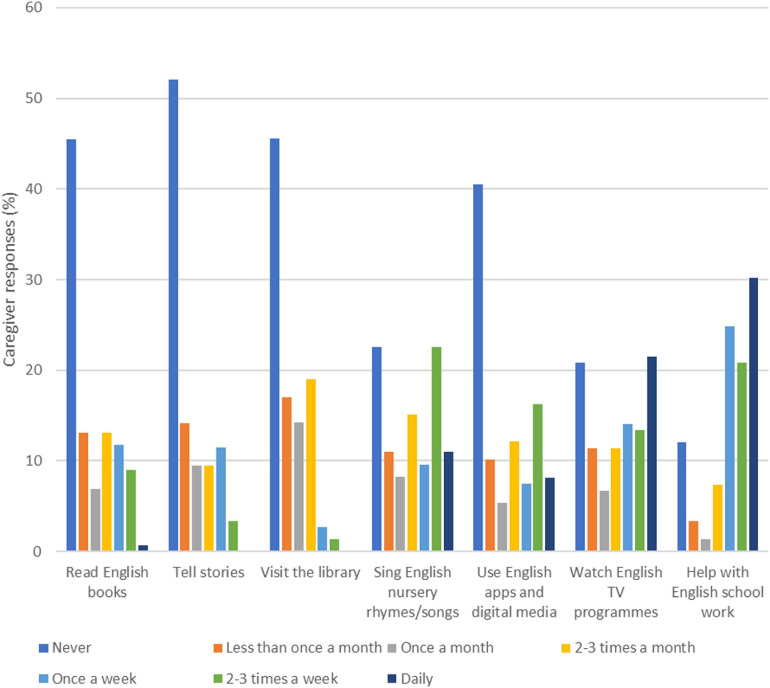
Proportion of caregivers reporting engaging in English activities with their child, by frequency of engagement (*n* = 149).

**FIGURE 2:**
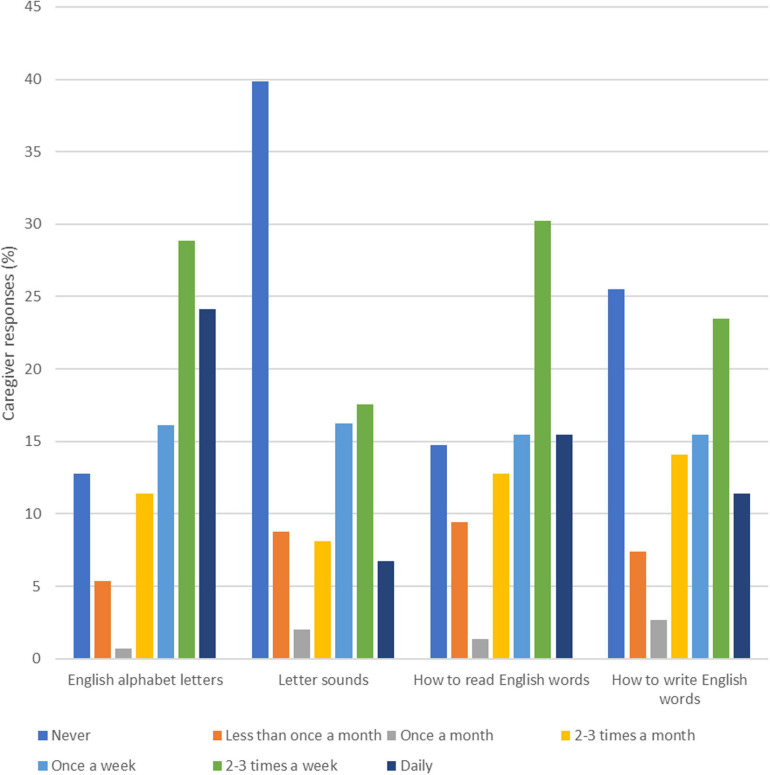
Proportion of caregivers reporting teaching printed-related skills in English to their child, by frequency of teaching (*n* = 149).

Figure 1 shows caregiver responses to a question about the frequency of engaging in English activities with their child. More than 40% of parents reported never reading English books, telling stories, visiting the library, or using English apps or digital media. Around 21% of caregivers read books and 15% told stories at least once a week as compared to 76% of caregivers helping their child with English schoolwork at the same frequency. Figure 2 shows responses to a question asking about how often caregivers teach their child print-related skills. The most common daily practice reported by caregivers was teaching English alphabet letters, with 24% of respondents reporting teaching alphabet letters daily, and 53% of respondents reporting teaching alphabet letters at least 2 to 3 times a week. By contrast, 40% of caregivers said they had never taught letter sounds.

Table 2 shows the final rotated 3 factor solution from an exploratory factor analysis (principal component factors) of caregiver responses to questions on the HLE, with loadings above 0.6 shown in bold. Factor 1 had high loadings on questions related to reading with children, including the age of the child when reading in English first commenced, highlighting and explaining vocabularies whilst reading, and telling stories in English. Factor 2 had high loadings on questions related to teaching children English letters and words. Factor 3 had high loadings on questions related to activities conducted with children, including singing songs, playing with apps, and watching TV programs. Factor 1 was therefore named “Shared reading and storytelling”, Factor 2 was named “Teaching of print-related skills”, and Factor 3 was named “Play and media-based activities”.

Correlations between key variables are shown in Table 3. The four measures of language and literacy were all positively correlated with each other (*r* = 0.32 to 0.46, *p*s < 0.05). Child age was positively correlated with all four measures of language and literacy, and with the measure of non-verbal intelligence (*p*s < 0.05). Gender was not significantly correlated with any of the variables (*p*s > 0.05). The measure of receptive vocabulary was positively correlated with the overall HLE variable and all 3 individual HLE factor variables (*p*s < 0.05), whilst the measure of phonological awareness was not significantly correlated with any of the HLE variables (*p*s > 0.05). All HLE variables were positively correlated with each other (*r* = 0.29 to 0.90, *p*s < 0.05), with the largest correlation between the overall HLE variable and the variable for factor 1 (shared reading and storytelling) (*r* = 0.90). OLS regressions demonstrated the proportion of variance in each of the four measures of language and literacy explained by (i) all control variables only (receptive vocabulary *R*^2^ = 0.24; phonological awareness *R*^2^ = 0.26; letter knowledge *R*^2^ = 0.19; word reading *R*^2^ = 0.24), and (ii) all control variables and the overall mean HLE variable combined (receptive vocabulary *R*^2^ = 0.24; phonological awareness *R*^2^ = 0.34; letter knowledge *R*^2^ = 0.24; word reading *R*^2^ = 0.27).

The results of four separate multilevel regressions are shown in Table 4, with each of receptive vocabulary, phonological awareness, letter knowledge, and word reading being the dependent variable in one of the four models, and the mean overall HLE variable being the independent variable in all four models. Mean overall HLE was positively associated with receptive vocabulary (β = 0.28, *p* < 0.001), letter knowledge (β = 0.22, *p* < 0.01), and word reading (β = 0.18, *p* < 0.05). Table 5 shows the results of the same set of four multilevel regressions as before, but this time using HLE factor 1 (Shared reading and storytelling) as the independent variable. All control variables and the level 2 variable were the same as before. Factor 1 (Shared reading and storytelling) was positively associated with receptive vocabulary (β = 0.23, *p* < 0.001), and word reading (β = 0.17, *p* < 0.05). Table 6 shows the results of the same set of four multilevel regressions as before, but this time using HLE factor 2 (Teaching of print-related skills) as the independent variable. Factor 2 (Teaching of print-related skills) was positively associated with receptive vocabulary (β = 0.15, *p* < 0.05), and letter knowledge (β = 0.24, *p* < 0.001). Similarly, Table 7 shows the results of four multilevel regressions using HLE factor 3 (Play and media-based activities) as the independent variable. Factor 3 (Play and media-based activities) was also positively associated with receptive vocabulary (β = 0.32, *p* < 0.001), and letter knowledge (β = 0.12, *p* < 0.01).

**TABLE 4:** Associations between mean overall HLE scores and 4 different measures of language and literacy.

	**Phonological awareness**			**Receptive vocabulary**			**Letter knowledge**			**Word reading**		
	***b***	**SE**	***p***	***b***	**SE**	***p***	***b***	**SE**	***p***	***b***	**SE**	***p***
**Fixed part**												
Age (months)	0.04	0.01	<0.001	0.00	0.01	0.692	0.03	0.01	<0.001	0.03	0.01	0.006
Non-verbal IQ (SD)	0.13	0.10	0.174	0.15	0.10	0.156	0.20	0.05	<0.001	0.08	0.13	0.548
Gender (girl)	–0.13	0.18	0.482	0.15	0.09	0.082	–0.12	0.12	0.289	–0.30	0.12	0.017
English at home (binary)	0.35	0.15	0.022	0.52	0.26	0.043	–0.04	0.23	0.860	–0.04	0.24	0.883
English extracurricular (binary)	0.03	0.26	0.893	0.24	0.15	0.108	0.11	0.12	0.319	–0.14	0.11	0.183
SES composite (SD)	0.17	0.07	0.012	0.14	0.07	0.046	0.00	0.13	0.996	0.05	0.10	0.576
English time at school (SD)	–0.09	0.08	0.251	0.20	0.12	0.112	–0.04	0.08	0.636	0.36	0.11	0.001
Mean overall HLE	0.07	0.10	0.459	0.28	0.07	<0.001	0.22	0.07	0.003	0.18	0.08	0.025
Constant	0.01	0.15	0.959	–0.15	0.16	0.341	0.05	0.11	0.681	0.21	0.08	0.010
**Random part**												
English time at school (SD)	0.00	-		0.00	0.05		0.00	-		0.22	0.07	
Kindergarten (SD)	0.00	-		0.27	0.09		0.00	-		0.00		
Residual (SD)	0.87	-		0.77	0.06		0.87	-		0.82	0.14	

*Results are from 4 different multilevel regressions, with each of 4 different standardized measures of language and literacy as the dependent variable in each model. All models included the standardized mean overall HLE score as the independent variable, and controlled for age, non-verbal IQ, gender, whether English was the primary language at home, whether the child had extracurricular English lessons, SES, and amount of English exposure at school. Kindergarten was the level 2 variable. “-” indicates that it was not possible to calculate SEs due to random estimates being very close to 0.*

**TABLE 5:** Associations between mean HLE factor 1 scores (Shared reading and storytelling) and 4 different measures of language and literacy.

	**Phonological awareness**			**Receptive vocabulary**			**Letter knowledge**			**Word reading**		
	***b***	**SE**	***p***	***b***	**SE**	***p***	***b***	**SE**	***p***	***b***	**SE**	***p***
**Fixed part**												
Age (months)	0.04	0.01	<0.001	0.00	0.01	0.752	0.03	0.00	<0.001	0.03	0.01	0.006
Non-verbal IQ (SD)	0.13	0.09	0.154	0.13	0.10	0.189	0.19	0.06	<0.001	0.07	0.12	0.598
Gender (girl)	–0.12	0.17	0.481	0.14	0.09	0.098	–0.13	0.11	0.258	–0.31	0.13	0.017
English at home (binary)	0.39	0.15	0.010	0.59	0.24	0.012	0.01	0.23	0.966	–0.01	0.24	0.977
English extracurricular (binary)	0.04	0.25	0.869	0.26	0.14	0.068	0.13	0.12	0.278	–0.13	0.11	0.234
SES composite (SD)	0.18	0.07	0.008	0.14	0.07	0.054	0.00	0.13	0.981	0.05	0.09	0.614
English time at school (SD)	–0.08	0.08	0.266	0.19	0.12	0.103	–0.04	0.08	0.629	0.35	0.11	0.001
HLE 1: Storytelling and story reading	0.02	0.09	0.837	0.23	0.06	<0.001	0.17	0.09	0.058	0.17	0.08	0.031
Constant	0.00	0.14	0.989	–0.16	0.15	0.287	0.04	0.11	0.731	0.21	0.08	0.008
**Random part**												
English time at school (SD)	0.00	-		0.00	0.00		0.00	-		0.22	0.07	
Kindergarten (SD)	0.00	-		0.26	0.10		0.00	-		0.00		
Residual (SD)	0.87	-		0.79	0.05		0.88	-		0.82	0.14	

*Results are from 4 different multilevel regressions, with each of 4 different standardized measures of language and literacy as the dependent variable in each model. All models included the standardized mean factor 1 HLE score as the independent variable, and controlled for age, non-verbal IQ, gender, whether English was the primary language at home, whether the child had extracurricular English lessons, SES, and amount of English exposure at school. Kindergarten was the level 2 variable. “-” indicates that it was not possible to calculate SEs due to random estimates being very close to 0.*

**TABLE 6:** Associations between mean HLE factor 2 scores (Teaching of print-related skills) and 4 different measures of language and literacy.

	**Phonological awareness**			**Receptive vocabulary**			**Letter knowledge**			**Word reading**		
	***b***	**SE**	***p***	***b***	**SE**	***p***	***b***	**SE**	***p***	***b***	**SE**	***p***
**Fixed part**												
Age (months)	0.03	0.01	<0.001	–0.01	0.01	0.174	0.03	0.01	<0.001	0.03	0.01	0.021
Non-verbal IQ (SD)	0.14	0.09	0.149	0.17	0.11	0.112	0.23	0.06	<0.001	0.09	0.13	0.473
Gender (girl)	–0.13	0.18	0.470	0.14	0.08	0.065	–0.15	0.11	0.182	–0.30	0.12	0.013
English at home (binary)	0.38	0.15	0.011	0.66	0.27	0.014	0.03	0.22	0.878	0.05	0.27	0.844
English extracurricular (binary)	0.03	0.26	0.895	0.24	0.18	0.177	0.10	0.12	0.415	–0.14	0.11	0.203
SES composite (SD)	0.19	0.07	0.010	0.18	0.06	0.005	0.05	0.13	0.675	0.08	0.09	0.340
English time at school (SD)	–0.08	0.08	0.290	0.24	0.15	0.126	–0.03	0.09	0.746	0.37	0.11	0.001
HLE 2: Teaching of print-related skills	0.05	0.06	0.397	0.15	0.06	0.015	0.24	0.04	<0.001	0.09	0.06	0.142
Constant	0.00	0.16	0.975	–0.16	0.17	0.355	0.05	0.11	0.616	0.20	0.09	0.017
**Random part**												
English time at school (SD)	0.00	-		0.24	0.27		0.00	-		0.24	0.13	
Kindergarten (SD)	0.00	-		0.22	0.29		0.00	-		0.00		
Residual (SD)	0.87	-		0.80	0.06		0.86	-		0.83	0.35	

*Results are from 4 different multilevel regressions, with each of 4 different standardized measures of language and literacy as the dependent variable in each model. All models included the standardized mean factor 2 HLE score as the independent variable, and controlled for age, non-verbal IQ, gender, whether English was the primary language at home, whether the child had extracurricular English lessons, SES, and amount of English exposure at school. Kindergarten was the level 2 variable. “-” indicates that it was not possible to calculate SEs due to random estimates being very close to 0.*

**TABLE 7:** Associations between mean HLE factor 3 scores (Play and media-based activities) and 4 different measures of language and literacy.

	**Phonological awareness**			**Receptive vocabulary**			**Letter knowledge**			**Word reading**		
	***b***	**SE**	***p***	***b***	**SE**	***p***	***b***	**SE**	***p***	***b***	**SE**	***p***
**Fixed part**												
Age (months)	0.04	0.01	<0.001	0.00	0.01	0.672	0.03	0.01	<0.001	0.03	0.01	0.009
Non-verbal IQ (SD)	0.14	0.10	0.163	0.17	0.11	0.100	0.22	0.06	<0.001	0.09	0.13	0.474
Gender (girl)	–0.10	0.19	0.596	0.22	0.09	0.009	–0.08	0.11	0.459	–0.26	0.12	0.024
English at home (binary)	0.35	0.15	0.017	0.55	0.23	0.018	0.06	0.21	0.790	0.01	0.23	0.956
English extracurricular (binary)	0.03	0.26	0.901	0.25	0.18	0.177	0.13	0.14	0.341	–0.14	0.11	0.221
SES composite (SD)	0.17	0.07	0.017	0.14	0.07	0.039	0.02	0.13	0.878	0.06	0.10	0.503
English time at school (SD)	–0.08	0.08	0.302	0.23	0.14	0.085	–0.02	0.09	0.773	0.37	0.10	< 0.001
HLE 3: Play and media-based activities	0.11	0.09	0.217	0.32	0.08	<0.001	0.12	0.04	0.005	0.15	0.08	0.070
Constant	0.00	0.16	0.976	–0.18	0.17	0.276	0.01	0.11	0.962	0.19	0.09	0.029
**Random part**												
English time at school (SD)	0.00	-		0.15	0.19		0.00	-		0.23	0.07	
Kindergarten (SD)	0.00	-		0.26	0.14		0.00	-		0.00		
Residual (SD)	0.87	-		0.76	0.06		0.89	-		0.83	0.14	

*Results are from 4 different multilevel regressions, with each of 4 different standardized measures of language and literacy as the dependent variable in each model. All models included the standardized mean factor 3 HLE score as the independent variable, and controlled for age, non-verbal IQ, gender, whether English was the primary language at home, whether the child had extracurricular English lessons, SES, and amount of English exposure at school. Kindergarten was the level 2 variable. “-” indicates that it was not possible to calculate SEs due to random estimates being very close to 0.*

Table 8 shows the results of four multilevel regressions, but this time including all three HLE factors as independent variables at the same time. After adjusting for the other two HLE factors: HLE factor 1 (Shared reading and storytelling) was positively associated with word reading (β = 0.13, *p* < 0.05); HLE factor 2 (Teaching of print-related skills) was positively associated with letter knowledge (β = 0.23, *p* < 0.001); and HLE factor 3 (Play and media-based activities) was positively associated with receptive vocabulary (β = 0.29, *p* < 0.001).

**TABLE 8:** Associations between mean HLE factor scores (all 3 factors included) and 4 different measures of language and literacy.

	**Phonological awareness**			**Receptive vocabulary**			**Letter knowledge**			**Word reading**		
	***b***	**SE**	***p***	***b***	**SE**	***p***	***b***	**SE**	***p***	***b***	**SE**	***p***
**Fixed part**												
Age (months)	0.04	0.01	<0.001	0.00	0.01	0.993	0.03	0.01	<0.001	0.03	0.01	0.008
Non-verbal IQ (SD)	0.15	0.10	0.133	0.16	0.10	0.118	0.21	0.06	<0.001	0.08	0.13	0.546
Gender (girl)	–0.09	0.18	0.612	0.21	0.09	0.018	–0.18	0.12	0.136	–0.29	0.13	0.026
English at home (binary)	0.36	0.15	0.013	0.52	0.23	0.022	–0.01	0.22	0.950	–0.03	0.24	0.886
English extracurricular (binary)	0.03	0.26	0.897	0.25	0.17	0.142	0.10	0.11	0.374	–0.14	0.11	0.217
SES composite (SD)	0.18	0.07	0.009	0.12	0.08	0.099	0.03	0.12	0.796	0.05	0.09	0.603
English time at school (SD)	–0.08	0.08	0.319	0.21	0.12	0.085	–0.04	0.09	0.670	0.36	0.10	0.001
HLE 1: Storytelling and story reading	–0.04	0.08	0.663	0.11	0.06	0.061	0.12	0.12	0.332	0.13	0.06	0.041
HLE 2: Teaching of print-related skills	0.00	0.04	0.985	–0.04	0.06	0.533	0.23	0.04	<0.001	0.01	0.10	0.957
HLE 3: Play and media-based activities	0.13	0.07	0.090	0.29	0.08	<0.001	–0.05	0.08	0.556	0.09	0.08	0.257
Constant	–0.01	0.15	0.936	–0.18	0.17	0.280	0.07	0.11	0.517	0.21	0.09	0.015
**Random part**												
English time at school (SD)	0.00	-		0.03	1.11		0.00	-		0.22	0.65	
Kindergarten (SD)	0.00	-		0.27	0.26		0.00	-		0.00		
Residual (SD)	0.86	-		0.75	0.06		0.86	-		0.82	0.39	

*Results are from 4 different multilevel regressions, with each of 4 different standardized measures of language and literacy as the dependent variable in each model. All models included all 3 standardized mean HLE factor scores as the independent variables, and controlled for age, non-verbal IQ, gender, whether English was the primary language at home, whether the child had extracurricular English lessons, SES, and amount of English exposure at school. Kindergarten was the level 2 variable. “-” indicates that it was not possible to calculate SEs due to random estimates being very close to 0.*

Sensitivity analysis was conducted by running all regressions once more but using mixed-effects Tobit models. The directionality of any significant association (positive or negative) between independent and dependent variables was consistent between all random slope multilevel regressions and all corresponding Tobit regressions. The presence or absence of statistical significance at the 5% level was also consistent, with the following exceptions. When using a Tobit model, HLE factor 1 (Shared reading and storytelling) was positively and significantly associated with letter knowledge (*b* = 1.51, *p* = 0.028); and HLE factor 3 (Play and media-based activities) was positively and significantly associated with phonological awareness (*b* = 0.67, *p* = 0.044), and with word reading (*b* = 2.70, *p* = 0.038), but not with letter knowledge (*p* > 0.05). When all three HLE factor variables were included as independent variables at the same time, results were consistent, with the exception that HLE factor 1 (Shared reading and storytelling) was not significantly associated with word reading (*p* > 0.05).

## Discussion

This study examined relations between multiple aspects of the HLE and the development of English as a second language among ethnic Chinese children in Hong Kong. It addressed three questions: (a) What kinds of home literacy practices are caregivers engaged in to support children’s English language and literacy development?; (b) What is the relationship between the HLE and children’s English language and literacy skills?; and (c) To what extent does the HLE predict children’s English language and literacy development? Our work captured the multifaceted nature of the HLE and examined a range of literacy activities and behaviors in predicting variability in early English language and literacy skills. The findings from this study extended current knowledge by providing new evidence on the HLE of children from different linguistic and cultural backgrounds and contributed to further understanding of the processes that support second language development across different contexts in the early years.

The present work revealed considerable variability in the types of literacy activities that caregivers were engaged in at home with their children. Two notable findings emerged: (1) a sizable portion of caregivers never read books, told stories, visited the library or used digital media to support children’s English language learning; and (2) the tendency for caregivers to teach children print-related skills and help with English schoolwork on a more frequent basis (i.e., at least once a week) was relatively higher than that of reading English books and telling stories with their children. These results suggest that in the context of Hong Kong, caregivers tend to prioritize formal literacy activities that are deemed related to school progress and achievement. Consistent with previous work that indicates Hong Kong parents’ demands for a rigorous academic curriculum to support children’s entry to primary school (e.g., Leung et al., 2013), the emphasis on print-related activities and schoolwork in the HLE reflect caregivers’ priorities in preparing children to meet academic requirements and excel in school. In turn, caregivers may not be as active in activities beyond schoolwork, such as telling stories or reading for pleasure with their children. The extent to which caregivers are involved in literacy activities in a second language, however, may be largely linked to their language proficiency levels (Dixon and Wu, 2014). For instance, as more complex language and vocabulary are found in children’s books than in adult conversations (Hayes and Ahrens, 1988), shared reading may require caregivers to possess a certain level of language proficiency in order to read the text and to engage in verbal exchanges with their children. Thus, the quantity and quality of shared reading may potentially be undermined by caregivers’ proficiency and confidence in English. Furthermore, while activity-based approaches have increasingly been implemented in English language teaching in kindergarten classrooms in Hong Kong, studies have also documented the use of traditional paper and pencil exercises and the emphasis on recognition of letters, sounds and words in the teaching and learning process (Lau and Rao, 2013; Ng and Rao, 2013). The value attached to formal approaches in language teaching in schools may potentially influence caregivers’ tendency to use more didactic approaches when exposing children to English at home and to target print-related skills rather than oral language skills in their interactions with children.

The results of this study indicated that the HLE was differentially related to children’s English language and literacy development. The overall HLE was positively correlated with receptive vocabulary, letter knowledge, and word reading. Specifically, shared reading and storytelling, as a factor, was correlated with receptive vocabulary and word reading; direct teaching of print-related skills (e.g., letter names and sounds) was correlated with receptive vocabulary and letter knowledge; and play and media-based activities (e.g., singing rhymes/songs, watching television programs) were correlated with receptive vocabulary. There were no significant correlations between any aspects of the HLE and phonological awareness. Multilevel regression analyses further confirmed the unique contribution of the HLE to children’s development of English as a second language regardless of children’s age, non-verbal IQ, gender, whether English was the primary language at home, whether there were extracurricular English lessons, SES backgrounds, and the amount of English exposure at school. Shared reading and storytelling contributed significantly to receptive vocabulary and word reading, and results were robust to sensitivity analysis. After the inclusion of all three factors in the same model simultaneously, shared reading and storytelling also significantly contributed to word reading. The findings are consistent with research evidence on the benefits of shared reading and storytelling on early language and literacy skills (Wood, 2002; Curenton et al., 2008; Evans and Shaw, 2008). Explicit teaching and coaching by adults (e.g., introducing and explaining vocabulary, helping children decode words, drawing attention to letter names and sounds), as well as provision of opportunities for children’s active participation during the reading process (e.g., adults prompting children to talk about the book) enable children to be exposed to varied vocabulary and elaborate forms of language (Whitehurst et al., 1988; Justice et al., 2005). In this study, a composite measure of shared reading and storytelling was used which included the frequency of shared reading, age of onset of reading, number of books at home, and reading behavior (i.e., verbal interactions during reading) and storytelling. It was, therefore, unclear whether the positive relation to receptive vocabulary and word reading was primarily due to aspects of shared reading or storytelling, or both. Further research will be needed to delineate the specific impacts of shared reading and storytelling on children’s language and literacy development. Nonetheless, the current study provides preliminary evidence suggesting that both the quantity and quality of shared reading, as well as storytelling play important roles in fostering children’s vocabulary and word reading skills in a second language.

Direct teaching of print-related skills predicted children’s receptive vocabulary and letter knowledge, and results were robust to sensitivity analysis. While past research found that the primary impact of parental teaching of print-related skills is on code-based skills, such as letter knowledge and phonological awareness (e.g., Evans et al., 2000; Sénéchal and LeFevre, 2002), the findings in this study supported the relations with receptive vocabulary skills as well. One plausible explanation for this association is the caregivers’ interaction style during print-focused activities. It is possible that caregivers may introduce new words while discussing about letter sounds or talk with children about words when teaching reading and writing, which may facilitate children’s oral language development. However, when all three factors were included in the same model simultaneously, teaching of print-related skills predicted letter knowledge only, suggesting that after controlling for letter knowledge, associations between teaching of print-related skills and receptive vocabulary were no longer significant. Another explanation may therefore be that receptive vocabulary and letter knowledge skills are related but teaching of print-related skills is more directly relevant for letter knowledge than receptive vocabulary. Further research is warranted into the mechanisms through which caregivers teach print-related skills and the verbal interactions that occur during print-focused activities. Further, our study did not find significant associations between any aspects of the HLE and phonological awareness skills. While there may be other mechanisms underlying the lack of association between HLE and phonological awareness (Sénéchal, 2006; Sénéchal and LeFevre, 2014), it may be the case that the HLE alone may not be sufficient in facilitating change in children’s development of phonological skills. Given the differences in the phonological features and orthographies between children’s L1 (Chinese) and L2 (English), children may specifically require explicit instruction both at home and at school, and frequent and varied exposure in different contexts to develop phonological skills in a second language.

Play and media-based activities contributed significantly to receptive vocabulary and letter knowledge, although only the contribution to receptive vocabulary was robust to sensitivity analysis. Specifically, play and media-based activities was a stronger predictor of children’s receptive vocabulary skills than either of the other HLE factors, and also compared to the overall HLE. When all three factors were included in the model simultaneously, play and media-based activities predicted receptive vocabulary only. These findings support previous research which documented positive links between individual or composite measures of activities other than shared reading and parental teaching of print-related skills and children’s oral language and/or code-related skills (Passenger et al., 2000; Levy et al., 2006; Uchikoshi, 2006). This study points to the importance of adopting a broad conceptualization of the HLE to facilitate a more comprehensive understanding of the range of home literacy experiences that may contribute to early language and literacy development. The inclusion of an array of literacy-related activities in the measure of the HLE may be particularly important in second language and/or multilingual contexts. There is a likelihood that caregivers who are not fully fluent in the second language may utilize audio-visual materials as additional sources of language exposure to children. Indeed, in our study, the tendency for caregivers to sing English nursery rhymes/songs, use English digital media and watch English television programs with children on a more frequent basis (i.e., at least once a week) was relatively higher than that of shared reading and storytelling. It is possible that caregivers rely on readily available audio-visual materials to serve as language models for children’s second language development. It is, however, unknown whether and to what extent caregivers are involved with their children during singing, television viewing and the use of digital media. As current research evidence suggests, children learn languages better from live social interactions than from screens alone (e.g., Roseberry et al., 2009). Future studies can consider examining the interactions between caregivers and children when activities, such as television viewing and use of digital media, are included as measures of the HLE.

Taken together, this study corroborates previous findings concerning the importance of active home literacy activities (i.e., caregivers’ efforts to directly engage children in literacy activities) (Burgess et al., 2002). As Sylva et al. (2004) concluded, the quality of interaction between caregivers and children is a more significant predictor of children’s outcomes than family background characteristics, such as income and education. There is thus, a need to enhance caregivers’ knowledge, skills and attitude in enriching the HLE and to mobilize resources to support caregivers in facilitating children’s language and literacy development. Prior studies have demonstrated the effectiveness of family literacy interventions that are aimed at developing parents’ capacity to engage children in literacy activities (Zevenbergen and Whitehurst, 2003; Sénéchal and Young, 2008; Manz et al., 2010; van Steensel et al., 2011). Practitioners, policymakers and researchers can capitalize on the potential of family literacy programs to address compelling issues surrounding children’s development of English as a second language. In a 12-week intervention program on parent-child reading in English in Hong Kong, children in the intervention group made gains in both English word reading and phonological awareness skills, suggesting the effectiveness of dialogic reading on second language development among ethnic Chinese children (Chow et al., 2010). Early childhood education programs that encourage school-based and home-based family engagement practices and have family engagement as a core component of their policies can further support children’s language and literacy development (Goodall and Vorhaus, 2011). For instance, schools that provide workshops on specific strategies for literacy improvement (e.g., reading strategies) or design curricula that connect home and school practices (e.g., extended learning activities at home) may promote involvement in children’s education and enable caregivers to develop the competencies to support their children. Indeed, caregivers are more likely to be involved in schools and at home when they recognize the importance of their roles in children’s learning, feel capable of assisting their children and feel invited by the school and their children (Hoover-Dempsey and Sandler, 1997). Further, public campaigns or community events that strengthen family and public participation in literacy activities may help support the development of children’s language and literacy skills. Particularly, community efforts to provide books, as well as support on home literacy activities for families from disadvantaged backgrounds can increase parent-child engagement at home (Odom et al., 2012).

It should be noted that there are several limitations to this study. First, caregivers’ self-reports of their engagement in home literacy practices may be subject to social desirability bias. Future studies can consider supplementing survey data with direct observations of literacy interactions or interviews with caregivers. Respondents to the caregiver survey were also not always the child’s primary caregiver, so interviewing primary caregivers or using direct observations could be helpful to triangulate across several data sources. Second, this study mainly examined the frequency of caregivers’ engagement in literacy activities as a measure of the HLE in predicting early language and literacy outcomes. It would be valuable to examine additional aspects of the HLE that have been found to explain variability in language and literacy development, such as parental beliefs and attitudes about literacy (Debaryshe, 1995; Weigel et al., 2006), parent-child interactions, such as maternal responsiveness and sensitivity (de Jong and Leseman, 2001; Tamis-Lemonda et al., 2001), parental modeling of reading behavior (Burgess et al., 2002), child literacy interest (Baroody and Diamond, 2012; Carroll et al., 2019), and parents’ and children’s foreign language reading anxiety (Chow et al., 2017). Third, this study did not consider the home literacy practices and development of children in the first language. Such data may contribute to more refined understanding of the HLE across languages and may yield important findings on the impact of the HLE on first and second language development. Fourth, while this study considered children’s exposure to English at home (whether English was the primary language), we did not have in-depth information about the circumstances under which English is spoken. More detailed information about the extent of children’s exposure to English, including language use of the child and each family member in the household may enable a more comprehensive understanding of the home language environment. Finally, this study only accounted for amount of exposure to English lessons in schools when analyzing the prediction of the HLE on children’s English language and literacy outcome. Future research can examine the quality of English language teaching in schools to further disentangle the processes that explain the effects of the HLE on children’s development of English as a second language.

## Conclusion

This study highlights variability in the home literacy practices of ethnic Chinese families in Hong Kong and demonstrates that aspects of the HLE are differentially related to children’s English vocabulary, phonological awareness, letter knowledge, and word reading skills. The present work provides a more nuanced understanding of the characteristics and influences of the HLE in the development of English as a second language in a multilingual context. It adds to a growing body of knowledge that points to the significant role of the HLE in children’s language and literacy skills and has the potential to inform policies and programs that promote family literacy practices. The findings from this study can serve as a basis for future cross-cultural comparisons of the HLE and the development of English as a second language among young children.

## Data Availability Statement

The raw data supporting the conclusions of this article will be made available by the authors, without undue reservation.

## Ethics Statement

The studies involving human participants were reviewed and approved by Human Research Ethics Committee, The University of Hong Kong. Written informed consent to participate in this study was provided by school principals, teachers and the participants’ legal guardian/next of kin.

## Author Contributions

CL conceptualized and implemented the study. BR conducted the statistical analysis. Both authors contributed to the article and approved the submitted version.

## Conflict of Interest

The authors declare that the research was conducted in the absence of any commercial or financial relationships that could be construed as a potential conflict of interest.
